# Supplementary material for: Association between oral microbiome and seven types of cancers in East Asian population: a two-sample Mendelian randomization analysis
Source: Front Mol Biosci. 2023 Nov 21;10:1327893. doi: 10.3389/fmolb.2023.1327893 (PMC10702768; doi:10.3389/fmolb.2023.1327893)
Supplement: Supplementary file 1 [file DataSheet1.ZIP › Supplementary Materials/MR plots of saliva/saliva═╝/colorectal cancer/pheno.1386_to_colorectal cancer_forest.pdf]

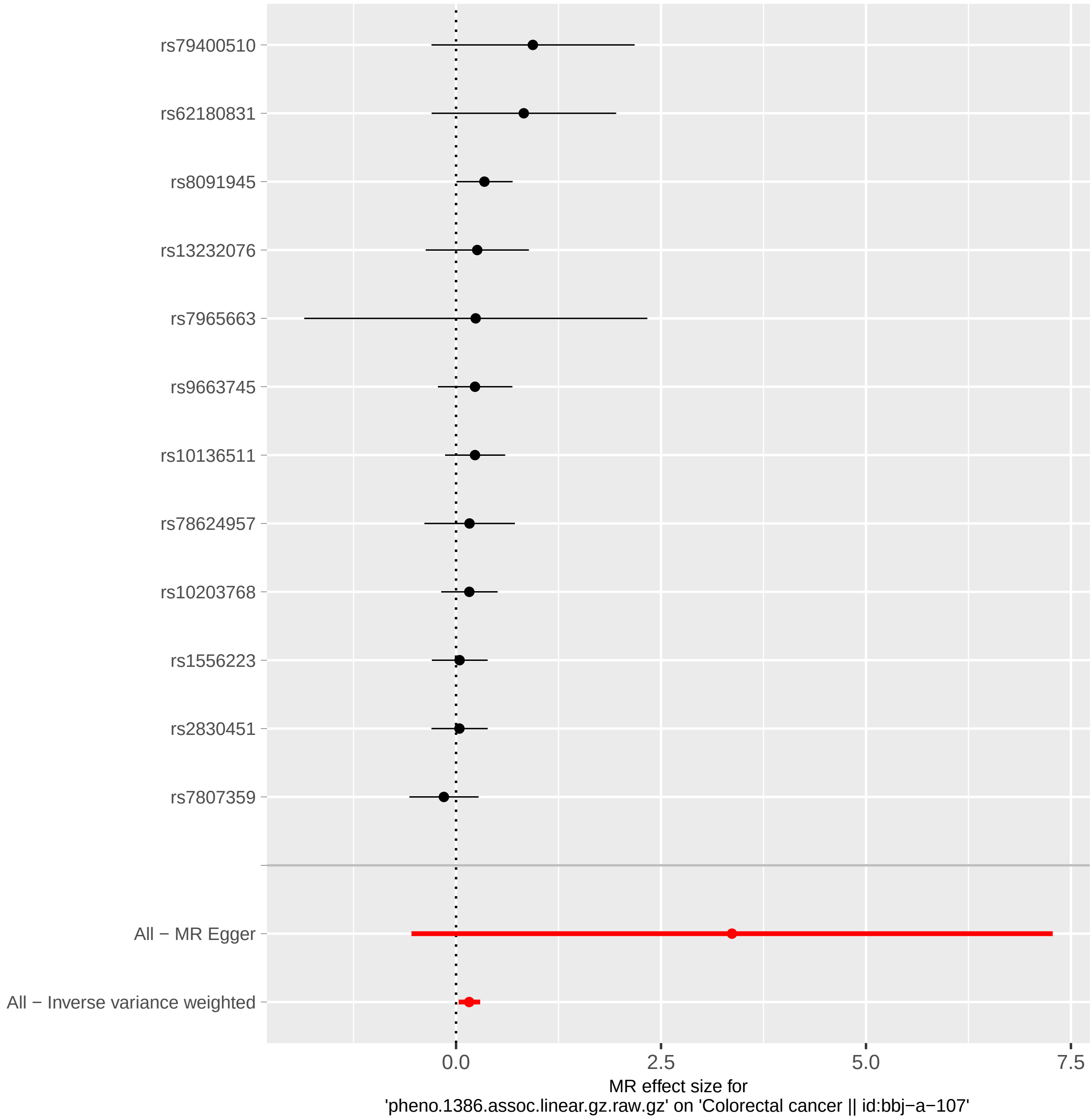

rs79400510
rs62180831
rs8091945
rs13232076
rs7965663
rs9663745
rs10136511
rs78624957
rs10203768
rs1556223
rs2830451
rs7807359
All − MR Egger
All − Inverse variance weighted
0.0
2.5
5.0
7.5
MR effect size for
'pheno.1386.assoc.linear.gz.raw.gz' on 'Colorectal cancer || id:bbj−a−107'
